# A 20-Year Antifungal Susceptibility Surveillance (From 1999 to 2019) for *Aspergillus* spp. and Proposed Epidemiological Cutoff Values for *Aspergillus fumigatus* and *Aspergillus flavus*: A Study in a Tertiary Hospital in China

**DOI:** 10.3389/fmicb.2021.680884

**Published:** 2021-07-22

**Authors:** Xinyu Yang, Wei Chen, Tianyu Liang, JingWen Tan, Weixia Liu, Yi Sun, Qian Wang, Hui Xu, Lijuan Li, Yabin Zhou, Qiqi Wang, Zhe Wan, Yinggai Song, Ruoyu Li, Wei Liu

**Affiliations:** ^1^Department of Dermatology and Venereology, Peking University First Hospital, Beijing, China; ^2^National Clinical Research Center for Skin and Immune Diseases, Beijing, China; ^3^Research Center for Medical Mycology, Peking University, Beijing, China; ^4^Beijing Key Laboratory of Molecular Diagnosis on Dermatoses, Beijing, China

**Keywords:** *Aspergillus* spp., susceptibility surveillance, epidemiological cutoff values, non-wild-type, a single-center study

## Abstract

The emergence of resistant *Aspergillus* spp. is increasing worldwide. Long-term susceptibility surveillance for clinically isolated *Aspergillus* spp. strains is warranted for understanding the dynamic change in susceptibility and monitoring the emergence of resistance. Additionally, neither clinical breakpoints (CBPs) nor epidemiological cutoff values (ECVs) for *Aspergillus* spp. in China have been established. In this study, we performed a 20-year antifungal susceptibility surveillance for 706 isolates of *Aspergillus* spp. in a clinical laboratory at Peking University First Hospital from 1999 to 2019; and *in vitro* antifungal susceptibility to triazoles, caspofungin, and amphotericin B was determined by the Clinical and Laboratory Standards Institute (CLSI) broth microdilution method. It was observed that *Aspergillus fumigatus* was the most common species, followed by *Aspergillus flavus* and *Aspergillus terreus*. Forty isolates (5.7%), including *A. fumigatus*, *A. flavus*, *A. terreus*, *Aspergillus niger*, and *Aspergillus nidulans*, were classified as non-wild type (non-WT). Importantly, multidrug resistance was observed among *A. flavus*, *A. terreus*, and *A. niger* isolates. *Cyp51A* mutations were characterized for 19 non-WT *A. fumigatus* isolates, and TR_34_/L98H/S297T/F495I was the most prevalent mutation during the 20-year surveillance period. The overall resistance trend of *A. fumigatus* increased over 20 years in China. Furthermore, based on ECV establishment principles, proposed ECVs for *A. fumigatus* and *A. flavus* were established using gathered minimum inhibitory concentration (MIC)/minimum effective concentration (MEC) data. Consequently, all the proposed ECVs were identical to the CLSI ECVs, with the exception of itraconazole against *A. flavus*, resulting in a decrease in the non-WT rate from 6.0 to 0.6%.

## Introduction

*Aspergillus* species are saprophytic molds widely distributed throughout the environment and are easily transported in the air and inhaled into the airway due to the small size of spores (Chabi et al., 2015). *Aspergillus* spp. can cause many human diseases, ranging from non-invasive allergic bronchopulmonary aspergillosis (ABPA) and chronic pulmonary aspergillosis to invasive aspergillosis (IA), and their clinical manifestations and prognosis vary widely (Cadena et al., 2016). IA is a life-threatening opportunistic infection associated with high morbidity and mortality rates, occurring mainly in immunocompromised patients, such as those with organ transplants or hematological malignancy and those receiving certain types of chemotherapy or immunosuppression therapy (Verweij et al., 2016). *Aspergillus fumigatus* is the most common *Aspergillus* spp. causing infection in humans, accounting for 70–80% of cases. The incidences of infections with other species, such as *Aspergillus flavus*, *Aspergillus niger*, and *Aspergillus terreus*, have been increasing, especially in immunocompromised hosts (Richardson and Lass-Flörl, 2008).

Triazoles have a broad spectrum of *in vitro* antifungal activity against molds and are currently the first-line antifungals for the treatment of IA, including itraconazole (ITC), voriconazole (VRC), and posaconazole (POS). Caspofungin (CAS) and amphotericin B (AMB) are important therapeutic agents for the systemic treatment of refractory IA as well as empirical or prophylactic therapy (Walsh et al., 2008). Despite advances in IA treatment, the mortality rates remain high, especially in immunosuppressed hosts. Antifungal resistance development is one of the major threats (Verweij et al., 2016). Resistance has emerged during the past decade, and the prevalence is increasing in some areas of the world. Therefore, identifying strains of *Aspergillus* spp. with different susceptibilities has important implications for understanding the susceptibility trend and selecting the correct antifungal agents.

Antifungal susceptibility testing of *Aspergillus* spp. has been standardized by both the Clinical and Laboratory Standards Institute (CLSI) and European Committee on Antimicrobial Susceptibility Testing (EUCAST) (CLSI, 2017). Breakpoints are used to determine whether the microorganisms are susceptible or resistant to the tested antifungals, which is an important basis for clinicians to select antifungal agents for the treatment of pathogenic infections. Breakpoints include clinical breakpoints (CBPs) and epidemiological cutoff values (ECVs). Currently, CBPs based on the minimum inhibitory concentration (MIC) distributions, pharmacokinetic and pharmacodynamics (PK/PD) parameters, animal data, and clinical outcomes for molds have not been established by the CLSI except for the CBP of VRC for *A. fumigatus* (CLSI, 2020). In the absence of sufficient data such as (PK/PD) parameters and clinical outcomes, CBPs are unable to be established. ECVs are used to evaluate the susceptibility of strains, to distinguish wild-type (WT) strains from strains with decreased susceptibility or acquired resistance mechanisms, and to monitor resistance development. ECVs for AMB, ITC, VRC, POS, CAS, and *Aspergillus* spp. have been defined by the CLSI and EUCAST using the broth microdilution (BMD) method (Espinel-Ingroff et al., 2010, 2011a,b; CLSI, 2018).

In the present study, we analyzed the antifungal activities of AMB, triazoles, and CAS against an extensive, geographically diverse collection of 706 *Aspergillus* spp. isolates from a clinical laboratory in China collected from 1999 to 2019. Furthermore, we applied CLSI ECVs to detect the emergence of resistant isolates. More importantly, WT distributions and tentative ECVs for AMB, ITC, VRC, POS, and CAS against *A. fumigatus* and *A. flavus* were proposed for the first time based on the gathered MIC/minimum effective concentration (MEC) data obtained from 20 years of antifungal susceptibility surveillance in China.

## Materials and Methods

### Isolation and Identification of *Aspergillus* Species

This study was a retrospective laboratory-based study of *Aspergillus* spp. infections from April 1999 to December 2019. All clinical isolates of *Aspergillus* spp. collected consecutively from unique patients in various Chinese hospitals were preserved at the Research Center for Medical Mycology at Peking University First Hospital, Beijing, China. The date of specimen collection, the type, and isolation site of the specimen were also recorded. This research was a surveillance study and did not involve human subjects.

To ensure the accuracy of species identification, all clinical isolates of *Aspergillus* spp. were identified to the species level in the central laboratory by a combination of morphological characteristics, matrix-assisted laser desorption/ionization–time of flight mass spectrometry (MALDI-TOF MS), and sequence analysis of the internal transcribed spacer (ITS), β-tubulin, and calmodulin genes (Sugui et al., 2014).

### Antifungal Susceptibility Testing

*In vitro* antifungal susceptibility testing of AMB, ITC, VRC, POS, and CAS against *Aspergillus* spp. isolates was performed using the CLSI M38-A3 method for filamentous fungi (CLSI, 2017). The antifungals used were AMB, ITC, VRC, POS, and CAS (all from Harveybio Gene Technology Co. Ltd., Beijing, China). The MIC was read at 48 h as the lowest concentration inhibiting visible growth for AMB, ITC, VRC, and POS. The MEC for CAS was defined at 24 h as the lowest concentration causing the growth of small, rounded, compact hyphal forms as compared with the hyphal growth seen in the growth control well. According to the recommendations in CLSI document M38-A3, the *Candida parapsilosis* ATCC 22019 and *Candida krusei* ATCC 6258 strains were used as quality control strains. The susceptibilities to AMB, ITC, VRC, POS, and CAS of *Aspergillus* spp. were evaluated, and the MICs/MECs were determined in this study. MIC/MEC ranges, MIC_50_/MEC_50_ (MIC causing inhibition of 50% of the isolates), and MIC_90_/MEC_90_ (MIC causing inhibition of 90% of the isolates) were also calculated. Because the CLSI has not established CBPs for *Aspergillus* species except the CBP of VRC for *A. fumigatus* (CLSI, 2020), CLSI ECVs were applied to classify the isolates as WT or non-wild type (non-WT) in terms of their antifungal susceptibilities (CLSI, 2018).

### Cyp51A Gene Sequencing of Triazole-Resistant *Aspergillus fumigatus* Isolates

Genomic DNA of non-WT *A. fumigatus* strains was extracted using a Biospin Fungus Genomic DNA Extraction Kit (BioFlux, Beijing, China) following the manufacturer’s instructions. The full sequences of the *cyp51A* gene with its promoter regions of non-WT *A. fumigatus* isolates were amplified using previously described PCR primers (Supplementary Table 1). The amplified products were sent to the BGI Company (Beijing, China) for sequencing. The DNA sequences of non-WT *A. fumigatus* isolates were aligned with those of the *A. fumigatus* reference strain (GenBank accession AF338659) using Clustal Omega.^1^

### Definition of Proposed Epidemiological Cutoff Values

The highest WT MIC/MEC is defined as ECV, which should be established by statistical techniques (Turnidge et al., 2006) or conventional methods (Espinel-Ingroff et al., 2010, 2011a,b). Briefly, the modeled WT population established by the statistical method is based on fitting a normal distribution starting at the lower end of the MIC range and calculating the mean and standard deviation (SD) of the cumulative normal distribution. These values are used to estimate the ECVs that capture at least 95% of the modeled WT population (Espinel-Ingroff et al., 2010, 2011a,b). The conventional method, also known as the “eyeball” method, visually inspects the histograms of the MIC distribution for a single species. The “eyeball” method has been used widely to define the ECVs for triazoles and echinocandins (Espinel-Ingroff et al., 2010, 2011a,b). Importantly, ECVs defined by the CLSI must include MIC distributions (≥100 MIC results per species and antifungal agent) from multiple (≥3) independent laboratories. In this study, since the number of *A. fumigatus* and *A. flavus* isolates exceeded 100, the proposed ECVs for *A. fumigatus* and *A. flavus* were established by combining the statistical calculation and “eyeball” method in our single-center laboratory.

### Statistical Analysis

All comparisons were performed using SPSS software version 18.0 (SPSS Inc., Chicago, IL, United States). Comparisons of continuous variables were performed using the Mann–Whitney test, and categorical variables were analyzed using the χ^2^ test or Fisher’s exact test. A *p*-value ≤ 0.05 was considered statistically significant.

## Results

### Specimen Origin and Species Distribution of *Aspergillus* Isolates

A total of 706 non-duplicate *Aspergillus* spp. isolates from individual patients were preserved at the Research Center for Medical Mycology in Peking University First Hospital from April 1999 to December 2019. Of these, 688 *Aspergillus* strains were isolated from various clinical sources, while the sources of the remaining 18 isolates were unknown. Regarding specimen types, over 50% of the *Aspergillus* spp. isolates (416/706, 58.9%) were recovered from sputum, 13.6% (96/706 isolates) were from bronchoalveolar lavage fluid (BALF), 9.2% (65/706 isolates) were from ear canal secretions, 5.1% (36/706 isolates) were from sinus secretions, 4.2% (30/706 isolates) were from biopsy tissues, 2.7% (19/706 isolates) were from body fluids (pleural fluid, ascetic fluid, and pericardial effusion), and 2.4% (17/706 isolates) were from the skin. Nine isolates were obtained from other body sites, including blood, urine, maxillary sinuses, and stool, accounting for no more than 5% (Table 1).

**TABLE 1:** *Aspergillus* spp. isolates recovered from clinical samples.

***N* (%)**	**Sputum**	**BALF**	**Ear canal**	**Sinus secretions**	**Tissues**	**Fluid**	**Skin (scales, pus)**	**Urine**	**Maxillary sinuses secretion**	**Blood**	**Stool**	**Unknown source**	**Total**
*Aspergillus fumigatus*	300 (72.1)	62 (64.6)	0	23 (63.9)	20 (66.7)	18 (94.8)	6 (35.3)	3 (100)	3 (100)	2 (100)	1 (100)	7 (38.8)	445
*Aspergillus flavus*	91 (21.9)	26 (27.1)	14 (21.5)	13 (36.1)	7 (23.3)	0	7 (41.2)	0	0	0	0	8 (44.4)	166
*Aspergillus terreus*	15 (3.6)	3 (3.1)	27 (41.5)	0	1 (3.3)	0	1 (5.9)	0	0	0	0	1 (5.5)	48
*Aspergillus niger*	5 (1.2)	2 (2.1)	23 (35.4)	0	2 (6.6)	0	1 (5.9)	0	0	0	0	2 (11.1)	35
*Aspergillus nidulans*	4 (0.9)	2 (2.1)	0	0	0	1 (5.2)	1 (5.9)	0	0	0	0	0	8
Other rare species^a^	1 (0.2)	1 (1.0)	1 (1.5)	0	0	0	1 (5.9)	0	0	0	0	0	4
Total	416 (58.9)	96 (13.6)	65 (9.2)	36 (5.1)	30 (4.2)	19 (2.7)	17 (2.4)	3 (0.4)	3 (0.4)	2 (0.3)	1 (0.1)	18 (2.5)	706

*BALF, bronchoalveolar lavage fluid.*

*^*a*^Other rare species included each isolate of *Aspergillus sydowii* (from ear canal), *Aspergillus penicillioides* (from cutaneous pus), *Aspergillus tamarii* (from sputum), and *Aspergillus undagawae* (from BALF).*

Among 706 *Aspergillus* spp. isolates, the proportion of *A. fumigatus* (385/548 isolates, 70.3%) isolated from respiratory tract samples (sputum, BALF, and sinus secretions) was significantly higher than that of isolates recovered from other specimen types (χ^2^ = 54.8, *p* < 0.05). *A. terreus* (27/65 isolates, 41.5%) and *A. niger* (23/65 isolates, 35.4%) isolated from ear canal secretions accounted for a higher proportion than isolates recovered from other specimen types (*p* < 0.05). More specifically, *A. fumigatus* accounted for the majority of strains (18/19 isolates, 94.8%) isolated from body fluids (ascetic fluid, pleural fluid, and pericardial effusion) and was the only species isolated from urine, maxillary sinus secretions, blood, and stool (Table 1).

*A. fumigatus* was the most predominant species (445/706 isolates, 63.0%), and *A. flavus* (166/706 isolates, 23.5%) was the second most common species, followed by *A. terreus* (48/706 isolates, 6.7%), *A. niger* (35/706 isolates, 5.0%), and *Aspergillus nidulans* (8/706 isolates, 1.1%). The remaining four species were cryptic species, including each isolate of *Aspergillus sydowii*, *Aspergillus penicillioides*, *Aspergillus tamarii*, and *Aspergillus undagawae*; and the prevalence rates were collectively <1%. Additionally, cryptic species were isolated in the past 5 years.

### Susceptibility to Triazoles, Amphotericin B, and Caspofungin

The antifungal activities of ITC, VRC, POS, AMB, and CAS against the 706 *Aspergillus* spp. isolates; and MIC/MEC_50_, MIC/MEC_90_, MIC/MEC ranges, and CLSI ECVs for the five agents tested against *Aspergillus* spp. isolates are presented in Table 2. A total of 666 isolates (94.3%) were susceptible to all tested antifungals. Since the CLSI M38-A3 method has not established ECVs for cryptic species, WT or non-WT strains were classified using CLSI ECVs at the *Aspergillus* species complex level (Won et al., 2018), and four cryptic isolates were susceptible to all antifungals tested.

**TABLE 2:** *In vitro* susceptibilities of the 706 *Aspergillus* spp. isolates against five antifungal agents and proposed ECVs for *Aspergillus fumigatus* and *Aspergillus flavus* obtained using CLSI M38-A3 broth microdilution method.

**Species (*N*)**	**Antifungal agents**	**No. of isolates**													**MIC/MEC (μg/ml)^d^**			**CLSI ECV^e^ (μg/ml)**	**WT (%)**	**Non-WT**	**Calculated ECV^f^ (μg/ml)**	**WT^g^**
		**MIC/MEC (μg/ml)**																							
		**0.008**	**0.015**	**0.03**	**0.06**	**0.125**	**0.25**	**0.5**	**1**	**2**	**4**	**8**	**16**	**32**	**50**	**90**	**Range**					
*Aspergillus fumigatus* (445)	ITC			2	3	10	61	119	234				16		1	1	0.03–16	1	96.4	16 (3.6%)	1	96.4%
	VRC					15	101	232	88	4		2	3		0.5	1	0.125–16	1	98.0	9 (2.0%)	1	98.0%
	POS		1	27	69	220	77	39	5	1			6		0.125	0.5	0.015–16	0.5	97.3	12 (2.7%)	0.5	97.3%
	CAS	13	28	89	126	76	71	42							0.06	0.25	0.008–0.5	0.5	100	0	0.5	100%
	AMB					4	5	18	180	238					2	2	0.125–2	2	100	0	2	100%
*Aspergillus flavus* (166)	ITC		1	2	3	6	45	40	59	9	1				0.5	1	0.015–4	1	94.0	10 (6.0%)	2	99.4%
	VRC			1		7	39	75	39	3	1		1		0.5	1	0.03–16	2	98.8	2 (1.2%)	2	98.8%
	POS^a^		1	2	2	8	38	8							0.25	0.5	0.015–0.5	0.5	100	0	ND	ND
	CAS	36	20	17	31	32	22	7			1				0.06	0.25	0.008–4	0.5	99.4	1 (0.6%)	0.5	99.4%
	AMB					1	2	7	51	76	23		3	1	2	4	0.125–32	4	97.6	4 (2.4%)	4	97.6%
*Aspergillus terreus* (48)	ITC	2		3	7	5	6	1	19	4		1			0.5	2	0.008–8	2	97.9	1 (2.1%)	ND	ND
	VRC				1	10	24	8	4	1					0.25	1	0.06–2	2	100	0	ND	ND
	POS^b^		5	3	6	3	3								0.06	0.25	0.015–0.25	1	100	0	ND	ND
	CAS	12	3	8	11	11	3								0.06	0.125	0.008–0.25	0.25	100	0	ND	ND
	AMB						1	1	4	24	15	3			2	8	0.25–8	4	93.7	3 (6.3%)	ND	ND
*Aspergillus niger* (35)	ITC					1	1	13	13	3	4				1	4	0.125–4	4	100	0	ND	ND
	VRC					2	7	16	10						0.5	1	0.125–1	2	100	0	ND	ND
	POS^c^			1		4	6	7							0.25	0.25	0.03–0.5	2	100	0	ND	ND
	CAS	11	5	4	3	6	5		1						0.03	0.25	0.008–1	0.25	97.1	1 (2.9%)	ND	ND
	AMB						1	2	11	17		3			2	2	0.25–8	2	91.4	3 (8.6%)	ND	ND
*Aspergillus nidulans* (8)	ITC				1	2	5								ND	ND	0.06–0.25	1	100	0	ND	ND
	VRC				2	4	1	1							ND	ND	0.06–0.5	2	100	0	ND	ND
	POS			1	1	5	1								ND	ND	0.03–0.25	1	100	0	ND	ND
	CAS	2		5						1					ND	ND	0.008–2	0.5	87.5	1 (12.5%)	ND	ND
	AMB								4	3	2				ND	ND	1–4	4	100	0	ND	ND
*Aspergillus sydowii* (1)	ITC						1								ND	ND	ND	2	100	0	ND	ND
	VRC								1						ND	ND	ND	2	100	0	ND	ND
	POS					1									ND	ND	ND	1	100	0	ND	ND
	CAS		1												ND	ND	ND	0.25	100	0	ND	ND
	AMB									1					ND	ND	ND	2	100	0	ND	ND
*Aspergillus penicillioides* (1)	ITC						1								ND	ND	ND	ND	100	0	ND	ND
	VRC								1						ND	ND	ND	ND	100	0	ND	ND
	POS					1									ND	ND	ND	ND	100	0	ND	ND
	CAS	1													ND	ND	ND	ND	100	0	ND	ND
	AMB									1					ND	ND	ND	ND	100	0	ND	ND
*Aspergillus tamarii* (1)	ITC								1						ND	ND	ND	1	100	0	ND	ND
	VRC							1							ND	ND	ND	2	100	0	ND	ND
	POS							1							ND	ND	ND	0.5	100	0	ND	ND
	CAS	1													ND	ND	ND	0.5	100	0	ND	ND
	AMB								1						ND	ND	ND	4	100	0	ND	ND
*Aspergillus undagawae* (1)	ITC							1							ND	ND	ND	1	100	0	ND	ND
	VRC								1						ND	ND	ND	1	100	0	ND	ND
	POS							1							ND	ND	ND	0.5	100	0	ND	ND
	CAS			1											ND	ND	ND	0.5	100	0	ND	ND
	AMB									1					ND	ND	ND	2	100	0	ND	ND

*MIC_50/90_ was the MIC inhibiting the growth of 50 and 90% of the isolates, respectively.*

*ITC, itraconazole; VRC, voriconazole; POS, posaconazole; AMB, amphotericin B; CAS, caspofungin; MIC, minimum inhibitory concentration; MEC, minimum effective concentration; ECVs, epidemiological cutoff values; non-WT, non-wild type; ND, not determined.*

*^*a*^The susceptibilities of 59 *A. flavus* isolates were tested using the CLSI broth microdilution method.*

*^*b*^The susceptibilities of 20 *A. terreus* isolates were tested using the CLSI broth microdilution method.*

*^*c*^The susceptibilities of 18 *A. niger* isolates were tested using the CLSI broth microdilution method.*

*^*d*^MIC_50_/_90_ values were calculated for species with ≥10 isolates.*

*^*e*^The CLSI ECVs were recommended by CLSI M59 document.*

*^*f*^The calculated ECVs captured ≥97.5% of the statistically modeled population.*

*^*g*^The percentage of MICs less than the calculated ECVs.*

As shown in Table 2, for *A. fumigatus*, AMB was active against all isolates, and VRC exhibited more efficacy than ITC and POS. For *A. flavus*, VRC was more effective than ITC and AMB. For *A. terreus* and *A. niger*, triazoles were effective against 97.9 and 100% of strains, while AMB was active against 93.7 and 91.4% of strains, respectively.

### Resistance to Triazoles, Amphotericin B, and Caspofungin

**FIGURE 1:**
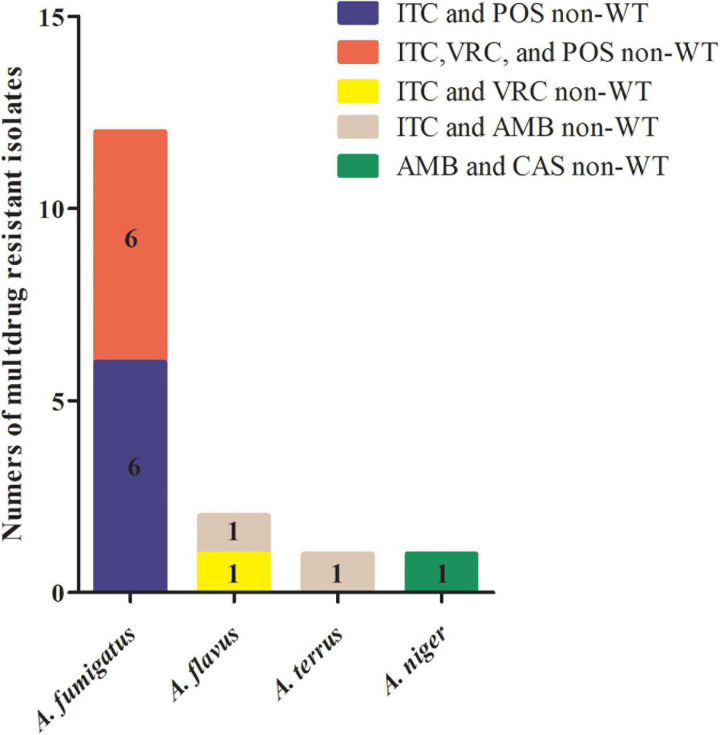
Multidrug-resistant *Aspergillus* spp. isolates collected in China from 1999 to 2019. Among 16 multidrug-resistant isolates of *Aspergillus* spp., six *Aspergillus fumigatus* isolates were cross-resistant to itraconazole (ITC) and posaconazole (POS); six *A. fumigatus* isolates were cross-resistant to ITC, voriconazole (VRC), and POS; one *Aspergillus flavus* isolate was cross-resistant to ITC and VRC; one *A. flavus* and one *Aspergillus terreus* isolate were co-resistant to ITC and amphotericin B (AMB); and one *Aspergillus niger* isolate was co-resistant to AMB and caspofungin (CAS).

A total of 40 isolates (5.7%) displayed a non-WT phenotype for at least one antifungal tested (Table 3). Of these, 16 isolates (40.0%) showed multidrug resistance to antifungals, and 75% (12/16) were *A. fumigatus* (Figure 1).

**TABLE 3:** The single and multidrug resistance of 706 *Aspergillus* spp. isolates to antifungals.

***Aspergillus* species (number of isolates tested)**	**Number (%) of isolates with resistance to antifungals**
*Aspergillus fumigatus* (445)	19 (4.3%)
ITC	4 (0.9%)
VRC	3 (0.7%)
ITC + POS	6 (1.3%)
ITC + VRC + POS	6 (1.3%)
*Aspergillus flavus* (166)	14 (8.4%)
ITC	7 (4.2%)
VRC	1 (0.6%)
CAS	1 (0.6%)
AMB	3 (1.8%)
ITC + VRC	1 (0.6%)
ITC + AMB	1 (0.6%)
*Aspergillus niger* (35)	3 (8.6%)
AMB	2 (5.7%)
AMB + CAS	1 (2.9%)
*Aspergillus terreus* (48)	3 (6.3%)
AMB	2 (4.2%)
ITC + AMB	1 (2.1%)
*Aspergillus nidulans* (8)	1 (12.5%)
CAS	1 (12.5%)

The prevalence of triazole resistance was 4.3% (19/445 isolates) in *A. fumigatus*, 6.0% (10/166 isolates) in *A. flavus*, and 2.1% (1/48 isolates) in *A. terreus* (Table 3). Additionally, four *A. flavus*, three *A. niger*, and three *A. terreus* exhibited non-WT phenotypes to AMB (Table 2). All *Aspergillus* spp. isolates were susceptible to CAS except three isolates: one *A. flavus* (MEC of 4 μg/ml) isolated in 2014, one AMB co-resistant *A. niger* (MEC of 1 μg/ml) isolated in 2013, and one *A. nidulans* (MEC of 2 μg/ml) isolated in 2019 (Table 2), suggesting that CAS resistance in clinical *Aspergillus* spp. isolates has emerged in recent years.

**FIGURE 2:**
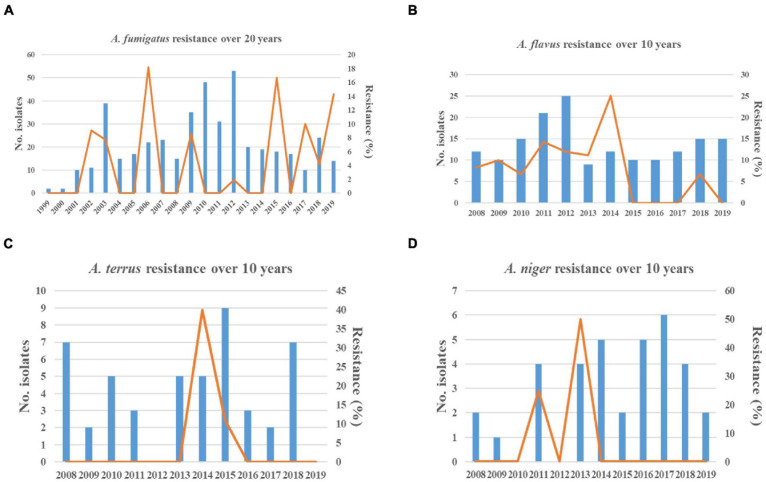
Resistance trends of *Aspergillus* spp. isolates collected over 20 years. Blue histograms: the total number of *Aspergillus* isolates collected each year. Yellow solid line: the frequency of resistant *Aspergillus* isolates each year. **(A)** The increasing trend in resistance of *Aspergillus fumigatus* was observed. **(B)** The decreasing trend in resistance of *Aspergillus flavus* was observed. **(C,D)** The trends in susceptibility of *Aspergillus terreus* and *Aspergillus niger* were stable.

As shown in Figure 2, the total isolates of *A. fumigatus* and *A. flavus* increased annually before 2012 but remained stable after 2012. Resistant strains of *A. fumigatus* and *A. flavus* were not isolated every year (Figure 2). Additionally, the 5-year proportion of resistant *A. fumigatus* isolates increased slightly from 5.06% (4/79 isolates) for 1999–2004 to 6.25% (7/112 isolates) for 2005–2009 and 8.43% (7/83 isolates) for 2015–2019 (*p* > 0.05). There was no statistically significant difference, while the increase in resistance for 2015–2019 compared with 2010–2014 (0.58%, 1/171 isolates) was statistically significant (χ^2^ = 8.859, *p* < 0.05). Although the triazole resistance rate of *A. fumigatus* decreased significantly from 6.25% for 2005–2009 to 0.58% for 2010–2014, the overall resistance trend increased slightly in the past 20 years. For *A. flavus*, the 5-year proportion of resistant *A. flavus* isolates decreased from 10.9% (10/92 isolates) for 2008–2013 to 5.4% (4/74 isolates) for 2014–2019 (Figure 2).

### Cyp51A Mutations in Non-wild Type *Aspergillus fumigatus* Isolates

Of the 19 non-WT *A. fumigatus* isolates, six harbored the G54W mutation, three harbored the G54R mutation, four exhibited the TR_34_/L98H/S297T/F495I mutation, three had the TR_46_/Y121F/T289A mutation, one carried the TR_34_/L98H mutation, and one carried the M220I mutation in the *cyp51A* gene. TR_34_/L98H/S297T/F495I was the most prevalent mutation. Surprisingly, there were no mutations in the *cyp51A* gene of one strain isolated in 2019 (Table 4).

**TABLE 4:** *Cyp51A* mutations of non-WT *A. fumigatus* isolates.

**Strains**	**GenBank accession numbers**	***cyp51A* mutation**	**MIC (μg/ml)**		
			**ITC**	**VRC**	**POS**
BMU02731	MW811158	M220I	16	1	0.25
BMU02810	MW811159	G54R	16	0.5	0.5
BMU02816	MW811160	G54R	16	0.5	0.5
BMU02998	MW814729	G54R	16	0.5	0.5
BMU03908	MW811161	G54W	16	0.5	16
BMU03941	MW811162	G54W	16	1	16
BMU03942	MW811163	G54W	16	1	16
BMU04053	MW814730	G54W	16	0.5	16
BMU04758	MW814731	G54W	16	1	16
BMU04835	MW811165	TR_34_/L98H	16	8	1
BMU04836	MW811166	TR_34_/L98H/S297T/F495I	16	2	1
BMU07160	MW814724	TR_34_/L98H/S297T/F495I	16	2	1
BMU07945	MW814725	TR_46_/Y121F/T289A	0.5	16	0.5
BMU07946	MW814726	TR_46_/Y121F/T289A	0.5	16	0.5
BMU08181	MW814727	TR_34_/L98H/S297T/F495I	16	2	1
BMU09386	MW811164	G54W	16	0.5	16
BMU09453	MW970052	TR_46_/Y121F/T289A	0.5	16	0.5
BMU09691	MW814728	TR_34_/L98H/S297T/F495I	16	2	1
BMU09765	MW788327	non-*cyp51A*	16	8	2

*BMU02731, BMU02810, BMU02816, BMU02998, BMU03908, BMU03941, BMU03942, BMU04053, and BMU04758 were isolated from one patient with pulmonary aspergilloma and chronic cavitary tuberculosis; BMU07945 and BMU07946 were isolated from one patient with ANCA-associated vasculitis; BMU04835 and BMU04836 were isolated from one patient with acute lymphoblastic leukemia. WT, wild type; MIC, minimum inhibitory concentration; ITC, itraconazole; VRC, voriconazole; POS, posaconazole; ANCA, anti-neutrophil cytoplasmic antibody.*

### The Establishment of Epidemiological Cutoff Values for *Aspergillus fumigatus* and *Aspergillus flavus*

Based on the statistical approach and ≥95% inclusion eyeball method used in our study, as shown in Table 2, the proposed ECVs for *A. fumigatus* and *A. flavus* isolates were calculated. Since only 59 isolates of *A. flavus* were examined for POS susceptibilities, the ECV of POS was not evaluated for *A. flavus*. Of note, the calculated ECVs were in agreement with the ECVs established by the CLSI in prior studies (CLSI, 2018), except the proposed ECV of *A. flavus* for ITC. Therefore, the susceptibility of *A. flavus* isolates to ITC was re-evaluated using the proposed ITC ECV listed in Table 2. Since the calculated ECV was 2 μg/ml, which was higher than the ECV recommended by the CLSI, the non-WT rate of *A. flavus* for ITC decreased from 6.0 to 0.6%.

## Discussion

In the present study, *A. fumigatus* was the most frequently isolated species among 706 *Aspergillus* strains, accounting for 63% of the isolates collected in this study, similar to the rates observed in several epidemiological studies in other countries (Krishnan et al., 2009; Alastruey-Izquierdo et al., 2013a). *A. flavus* was the second most commonly isolated species, which was in accordance with previous studies in the United States (Balajee et al., 2009), Europe (Alastruey-Izquierdo et al., 2013b), and Brazil (Negri et al., 2014). *A. terreus* and *A. niger* were the third and fourth most common isolates, respectively. However, *A. terreus* and *A. niger* were reported as the second most common species in Austria (Lackner et al., 2016) and Korea (Heo et al., 2015), respectively, indicating that there are geographical variations in the prevalence of different species. Additionally, sputum was the most common source of the clinically isolated *Aspergillus* spp. over the 20 years. *A. fumigatus* and *A. flavus* were mainly isolated from respiratory tract specimens. However, *A. terreus* and *A. niger* were mainly isolated from ear specimens.

The results of *in vitro* susceptibility testing listed in Table 2 showed that AMB was the most active against 100% of *A. fumigatus* isolates and that triazoles were effective against 95.7% of *A. fumigatus* isolates. In agreement with our finding, the susceptibility profiles of 159 clinical *A. fumigatus* isolates collected from different areas in China also showed that all isolates were susceptible to AMB and that 95.6% were susceptible to triazoles (Deng et al., 2017). A retrospective surveillance study in Portugal also indicated that AMB MICs of all *A. fumigatus* isolates were ≤2 μg/ml, while ITC, VRC, and POS were effective against 95.8, 97.4, and 84.7% of *A. fumigatus* isolates, respectively (Pinto et al., 2018). Gomez-Lopez et al. (2003) also demonstrated that AMB inhibited all *A. fumigatus* isolates at a concentration of 2 μg/ml. Likewise, the susceptibility result of a study from multiple hospitals in Shanghai, China, also showed that AMB was more effective than triazoles against *A. fumigatus* (Xu et al., 2020). Therefore, AMB appears to be the most active antifungal agent against *A. fumigatus*. This may be due to the fact that AMB is a polyene fungicidal agent with excellent property of high activity, while azole compounds inhibit fungal growth (Wiederhold, 2017). The fungicidal ability of AMB is higher than that of triazoles (Perlin et al., 2017). On the other hand, since the clinical use of AMB is limited due to nephrotoxicity, triazoles are recommended as the first-line therapy for IA, leading to the emergence of triazole resistance (Perlin et al., 2017; Wiederhold, 2017).

As shown in Table 4, the mutations in the *cyp51A* gene are the most common reasons conferring resistance to triazoles in *A. fumigatus*. Among 19 non-WT *A. fumigatus* strains, six types of the *cyp51A* mutations (M220I, G54W, G54R, TR_34_/L98H, TR_34_/L98H/S297T/F495I, and TR_46_/Y121F/T289A) were observed. The emergence of *A. fumigatus* harboring TR_34_/L98H/S297T/F495I mutation in China was first reported from a global surveillance study conducted in 2008 and 2009 (Lockhart et al., 2011). Chen et al. (2015) reported the first isolation of TR_46_/Y121F/T289A *A. fumigatus* strain from a Chinese patient without triazole exposure in 2015, which was related to two strains from clinical and environmental samples obtained in the Netherlands by genetic analysis based on microsatellite genotyping. Clinical and environmental *A. fumigatus* strains harboring TR_34_/L98H and TR_34_/L98H/S297T/F495I mutations were isolated in China; genetic typing and phylogenetic analysis showed that TR_34_/L98H isolates had a clonal expansion worldwide, while the TR_34_/L98H/S297T/F495I isolates harbored a distinct genetic background with resistant isolates from other countries (Chen et al., 2016). These mutations were also reported in many countries (Sharma et al., 2015; Chowdhary et al., 2017; Leonardelli et al., 2017). Additionally, one strain without mutations in the *cyp51A* gene was isolated in 2019 in this study. Recent studies have demonstrated the occurrence of triazole-resistant *A. fumigatus* without *cyp51A* gene mutations (Hagiwara et al., 2018; Sharma et al., 2019). Therefore, the strain may have non-*cyp51A*-mediated resistance mechanisms, and further research is worth conducting.

As shown in Table 2, triazoles were likely to be more effective than AMB against *A. flavus*, *A. terreus*, and *A. niger*. Since the total number of non-*fumigatus* strains isolated was low during the 10-year period and the susceptibility result of a single-center study was insufficient, it is difficult to consider triazoles as the potent agents against non-*fumigatus* strains, although the susceptibility result was in accordance with the previous study showing triazoles were the most active compounds against non-*fumigatus* species (Gomez-Lopez et al., 2003). However, the emergence of AMB resistance in *A. flavus* and *A. niger* strains should raise concerns. *A. terreus* is considered intrinsically resistant to AMB (Lass-Flörl et al., 2005; Blatzer et al., 2015).

As shown in Figure 2, the 5-year proportion of resistant *A. fumigatus* isolates was 5.06% for 1999–2004, 6.25% for 2005–2009, 0.58% for 2010–2014, and 8.43% for 2015–2019. The increase in resistance for 2015–2019 compared with 2010–2014 was statistically significant (χ^2^ = 8.859, *p* < 0.05). In various time periods, the prevalence of resistance was inconsistent and dynamic. Among 19 non-WT *A. fumigatus*, nine strains of BMU02731, BMU02810, BMU02816, BMU02998, BMU03908, BMU03941, BMU03942, BMU04053, and BMU04758, harboring the single point mutations in the *cyp51A* gene, were isolated from one patient with pulmonary aspergilloma and chronic cavitary tuberculosis before 2009 (Chen et al., 2005). The patient had a history of long-term ITC therapy (Chen et al., 2005). The presence of a cavity allows for asexual sporulation to occur; and with chronic azole exposure, numerous spontaneous mutations may occur in the conidia (Rivero-Menendez et al., 2016; Buil et al., 2019). Hence, due to ITC exposure, nine triazole-resistant strains were continuously isolated from the patient, resulting in high resistance rates in 1999–2004 (5.06%) and 2005–2009 (6.25%). Additionally, strains harboring TR_34_/L98H and TR_46_/Y121F/T289A were gradually isolated after 2009. Since TR_34_ or TR_46_ mutations are believed to be primarily driven by the use of azole fungicides in the environment (Chen et al., 2016; Rivero-Menendez et al., 2016), patients are infected by inhaling resistant conidia that already harbor azole resistance mechanisms under environmental exposure, presumably the consequence of exposure to azole fungicides used in agriculture (Chen et al., 2016; Rivero-Menendez et al., 2016).

Although the prevalence of resistance showed fluctuations, the overall trends toward decreasing susceptibility to triazoles were observed in *A. fumigatus* over the past 20 years. Previous studies were consistent with our finding. Nine-year susceptibility trends of 1,789 clinical *Aspergillus* spp. isolates obtained from more than 60 medical centers worldwide showed that the proportion of non-WT *A. fumigatus* isolates ranged from 0.7 to 4.0% for ITC, from 1.1 to 5.7% for POS, and from 0.0 to 1.6% for VRC (Pfaller et al., 2011). A 23-year continuous surveillance study for *A. fumigatus* in the Netherlands also observed an increasing trend of triazole resistance in clinical *A. fumigatus* isolates (Buil et al., 2019). Hence, the increasing prevalence of triazole resistance is a worldwide concern (Choukri et al., 2015; Chowdhary et al., 2017; Buil et al., 2019). In 2018, Chen et al. (2020) reported that 10.2% of *A. fumigatus* isolates collected from 63 soil samples from agricultural farms or greenhouses were azole resistant, and 18 resistant strains were cultured from soil samples acquired from strawberry fields. In a study in the United Kingdom, azole-resistant *A. fumigatus* isolates were identified in several products that originated from China, including tea and pepper (Chen et al., 2020). The total amount of azole fungicides used in agriculture accounted for more than one-third of fungicides used in China during 2013–2016 (Zhang et al., 2011). All these findings suggested that more resistant *A. fumigatus*, harboring the TR_34_ or TR_46_ mutations, were isolated from the environment in China. Similarly, azole-resistant *A. fumigatus* isolates have been recovered from the environment in an increasing number of countries (Choukri et al., 2015; Rivero-Menendez et al., 2016; Verweij et al., 2016; Perlin et al., 2017), with the frequency of azole resistance varying widely. Additionally, it has been reported that more resistant strains from China harbored the identical genetic background with resistant isolates from other countries by genetic analysis based on microsatellite genotyping (Chen et al., 2016; Deng et al., 2017), suggesting that the spread of resistant strains in the environment is increasing worldwide. Considering that the TR_34_/L98H was the most common type of mutation in this study, we speculated that the decreasing trend in susceptibility of *A. fumigatus* to triazoles shown in Figure 2 may be correlated with the increased exposure and transmission of resistant strains in the environment.

The possible reasons for the fluctuations in the prevalence of resistance in this study are as follows. Firstly, the number of resistant strains in the environment is increasing. Secondly, the triazole exposure in patients is increasing. Thirdly, the number of isolates collected was low, and the susceptibility result of a single-center study was limited. Finally, all isolates of *Aspergillus* spp. were recovered from clinical samples only.

Additionally, since only four isolates of *A. flavus* were non-WT to AMB, the trend in susceptibility of *A. flavus* to AMB seemed to be stable over the 10-year period, which was inconsistent with other studies showing that more *A. flavus* isolates were resistant to AMB (Gonçalves et al., 2013; Reichert-Lima et al., 2018; Rudramurthy et al., 2019). Given that *A. flavus* was the second leading species in our collection, continuous susceptibility surveillance will be still needed.

Cryptic species were found to account for over 10% of *Aspergillus* spp. clinical isolates and to be resistant to at least one of the antifungals available in Brazil, Spain, and Korea (Alastruey-Izquierdo et al., 2013a; Negri et al., 2014; Won et al., 2018). While only four isolates of cryptic species were identified, including *A. sydowii*, *A. penicillioides*, *A. tamarii*, and *A. undagawae*, and all were susceptible to the antifungals tested. Albeit the number of cryptic species was low in this study, attention should be paid to the emergence of resistant cryptic species.

Considering that the CBPs of *Aspergillus* spp. for common antifungals have not been determined except for the CBP of VRC for *A. fumigatus* (CLSI, 2020), the ECVs proposed for *A. fumigatus* and *A. flavus* were calculated. The proposed ECVs were in agreement with those defined by the CLSI except the ECV of ITC for *A. flavus* (Espinel-Ingroff et al., 2010, 2011a,b; CLSI, 2018). The susceptibility of *A. flavus* isolates to ITC was re-evaluated using the proposed ECV, showing that the prevalence of non-WT *A. flavus* isolates reduced significantly from 6 to 0.6%. The ECVs, established by CLSI using statistical methods combined with the “eyeball” method, must include MIC distributions (≥100 MIC results per species and antifungal agent) from multiple (≥3) independent laboratories (Espinel-Ingroff et al., 2010, 2011a,b), while the ECVs defined in this study were based on the gathered MIC data of strains collected from multiple geographic locations and preserved in a single center in China over 20 years. The proposed ITC ECV of *A. flavus* was higher than that defined by the CLSI, probably because the susceptibility of *A. flavus* isolates to ITC in China may be lower than that of other countries. On the other hand, the susceptibility result of a single-center study could not fully represent the susceptibility of *A. flavus* strains nationwide. Therefore, multicenter surveillance data in China need to be collected to establish accurate ECVs in the future.

In conclusion, this is the first 20-year retrospective surveillance study for clinically isolated *Aspergillus* spp. in China. Several species of *Aspergillus* spp. have developed drug resistance and even multidrug resistance, and the resistance mechanisms of non-WT *A. fumigatus* strains have been also described. Moreover, the proposed ECVs were established for *A. fumigatus* and *A. flavus*, which will be an essential step in developing CBPs and be useful for resistance surveillance in China. Hence, the significance of this study is to establish continuous susceptibility surveillance and determine antifungal susceptibility trends, providing a basis for clinical medication.

## Data Availability Statement

The datasets presented in this study can be found in online repositories. The names of the repository/repositories and accession number(s) can be found in the article/Supplementary Material.

## Author Contributions

XY, WC, TL, JT, WxL, YS, QW, HX, LL, YZ, QqW, and YgS contributed to the species identification and antifungal susceptibility testing. WL designed all the experiments of the study. XY, QW, and YZ contributed to molecular biology experiments. XY and WL contributed to writing and reviewing the manuscript. All authors read and approved the final manuscript.

## Conflict of Interest

The authors declare that the research was conducted in the absence of any commercial or financial relationships that could be construed as a potential conflict of interest.

## Footnotes

1 https://www.ebi.ac.uk/Tools/msa/clustalo/
