# Supplementary material for: A 20-Year Antifungal Susceptibility Surveillance (From 1999 to 2019) for Aspergillus spp. and Proposed Epidemiological Cutoff Values for Aspergillus fumigatus and Aspergillus flavus: A Study in a Tertiary Hospital in China
Source: Front Microbiol. 2021 Jul 22;12:680884. doi: 10.3389/fmicb.2021.680884 (PMC8339419; doi:10.3389/fmicb.2021.680884)
Supplement: Supplementary file 1 [file Table_1.DOCX]

| Table S1 Primers for *cyp51A* gene amplification and sequencing | | | |
| --- | --- | --- | --- |
| Primer name | **Function** | **Primer sequence (5′‐> 3′)** |  |
| P1 | Amplification and sequencing | GTCGATCTGTGTGACACCAC |  |
| P2 | Amplification and sequencing | GTTGGAATACATTCAATTAG |  |
| P3 | Sequencing | ATACTATGGCTTTCATATGT |  |
| P4 | Sequencing | TCCTCGAAATGGTGCCGATGC |  |
| P5 | Sequencing | AGAGTCTCATGTGCCACTTATTG |  |
| P6 | Sequencing | CACAGCCAAAAGTCCTCGAAG |  |
